# Adverse childhood experiences, psychosocial well-being and cognitive development among orphans and abandoned children in five low income countries

**DOI:** 10.1186/1472-698X-14-6

**Published:** 2014-03-10

**Authors:** Maya Escueta, Kathryn Whetten, Jan Ostermann, Karen O’Donnell

**Affiliations:** 1 Terry Sanford Institute of Public Policy, Duke University, Durham, NC, USA; 2 Center for Health Policy, Duke Global Health Institute, Duke University, 2812 Erwin Rd., Suite 403, Durham, North Carolina 27705, USA; 3 Department of Community and Family Medicine, Duke University, Durham, NC, USA; 4 Center for Child and Family Health, Duke University, Durham, NC, USA

**Keywords:** Orphans, Education, Trauma, Emotional difficulties, Cognition, POFO

## Abstract

**Background:**

Development policymakers and child-care service providers are committed to improving the educational opportunities of the 153 million orphans worldwide. Nevertheless, the relationship between orphanhood and education outcomes is not well understood. Varying factors associated with differential educational attainment leave policymakers uncertain where to intervene. This study examines the relationship between psychosocial well-being and cognitive development in a cohort of orphans and abandoned children (OAC) relative to non-OAC in five low and middle income countries (LMICs) to understand better what factors are associated with success in learning for these children.

**Methods:**

Positive Outcomes for Orphans (POFO) is a longitudinal study, following a cohort of single and double OAC in institutional and community-based settings in five LMICs in Southeast Asia and sub-Saharan Africa: Cambodia, Ethiopia, India, Kenya, and Tanzania. Employing two-stage random sampling survey methodology to identify representative samples of OAC in six sites, the POFO study aimed to better understand factors associated with child well-being. Using cross-sectional and child-level fixed effects regression analyses on 1,480 community based OAC and a comparison sample of non-OAC, this manuscript examines associations between emotional difficulties, cognitive development, and a variety of possible co-factors, including potentially traumatic events.

**Results:**

The most salient finding is that increases in emotional difficulties are associated with lags in cognitive development for two separate measures of learning within and across multiple study sites. Exposure to potentially traumatic events, male gender, and lower socio-economic status are associated with more reported emotional difficultiesin some sites. Being female and having an illiterate caregiver is associated with lower performance on cognitive development tests in some sites, while greater wealth is associated with higher performance. There is no significant association between orphan status *per se* and cognitive development, though the negative and significant association between higher emotional difficulties and lags in cognitive development hold across all orphan subgroups.

**Conclusions:**

These findings suggest that interventions targeting psychosocial support for vulnerable children, especially vis a vis traumatic experiences, may ease strains inhibiting a child’s learning. Family based interventions to stabilize socioeconomic conditions may help overcome psychosocial challenges that otherwise would present as barriers to the child’s learning.

## Background

The plight of orphans^a^ and abandoned children (OAC) is an increasing global problem that is particularly pervasive in Southeast Asia and sub-Saharan Africa [1]. Improving the educational attainment of the 153 million orphans and other vulnerable children worldwide is a key goal for development policymakers and practitioners. International declarations such as the Millennium Development Goals and the Education for All Movement indicate that the educational attainment of vulnerable children has become a global priority. Most recently, the 2011 Political Declaration on HIV/AIDS targeted increases in school attendance of orphans as an important and measureable indicator of progress [2].

To understand which policies can improve educational attainment for OAC, decision makers must first understand the determinants of and barriers to these outcomes. However, there are mixed results regarding which factors, including orphanhood itself, are significantly associated with educational attainment. Previous research shows that the loss of a parent can lead to a series of developmental disadvantages resulting in poor education [3-7], such as lags in grade for age and school attendance relative to non-orphans [3,7,8]. In contrast, other studies find little negative impact of parental death on child education [9-11] and instead find that alternative factors such as wealth, age, or the child’s relationship to the head of household are better predictors of education outcomes [12]. Importantly, many of these studies are restricted to single country analyses, rendering results arguably context specific.

Additionally, few studies examining the educational attainment of orphans move beyond outcomes such as school enrollment, grade for age, and attendance to disentangle what factors contribute to a child’s learning [13,14]. Other outcomes may provide more meaningful information. A recent study showed that tests of cognitive development can be a useful measure in understanding OAC learning and educational experience across settings. In particular, cognitive development was found to be positively associated with increases in exposure to formal education [15].

One factor that may offer some insight into the development of OAC is mental health. Previous research, though not focused on orphans, highlights the role of adverse childhood experiences in life outcomes [16]. Cumulative effects from multiple risk factors have been shown to be more predictive of compromised early cognitive development among vulnerable children than any one risk factor alone [17]. In the research on orphans, the role of psychosocial status on multiple outcomes has been studied, though not specifically in relation to learning outcomes. Research on OAC in low and middle income countries found that orphaned children are more susceptible than non-orphans to exposure to potentially traumatic events due to lack of adequate adult protection [18-21]. A recent study found that OAC anxiety and emotional difficulties increased with additional exposure to potentially traumatic events [22]. Another recent study found associations between orphan risk of psychosocial difficulties and subsequent risk of HIV infection [23].

While there is a clear need for mental health interventions among OAC, few investigators have examined the role of mental health in predicting OACs’ cognitive development [24-26]. To our knowledge, no study has attempted to do a cross-country analysis of community based OAC to address these questions. Through these analyses, we aim to understand better the linkages between emotional difficulties and a child’s ability to participate in and gain from education in a context where children are particularly vulnerable to adverse events and subsequent emotional difficulties. In this manuscript, we employ within-country and cross-country analyses to examine associations between exposure to adverse childhood events, the emotional difficulties that OAC face, and their cognitive development.

## Methods

### Study description

Positive Outcome for Orphans (POFO) is an ongoing longitudinal study following a cohort of children, starting ages 6 to 12, who live in institutional or community-based settings in 5 low income countries: Cambodia, Ethiopia, Kenya, India, and Tanzania. This analysis used 3 years of data from the community-based sample to address the relationships stated above.

### Study sample

The detailed sampling strategy and general characteristics of the sample have been reported elsewhere [26,27]. The following describes the elements of the sampling strategy applicable to this analysis.

The POFO study utilized a two stage random sampling methodology to identify a representative sample of 1,480 orphaned and abandoned children living in community-based settings in six sites across five low and middle income countries. Within each site, geographic or administrative boundaries were used to define sampling areas (clusters), 50 clusters were randomly selected at each site, and up to five eligible children ages 6-12 years were selected from each cluster. Eligible children were defined as follows: orphans were those children for whom one or both parents had died [1] and an abandoned child was one whose parents had left with no expectation of return. Eligible children were randomly selected from available lists or through a house-to-house census. One child per household was selected to participate in the study. For households with multiple age-eligible children, the child whose name started with the earliest letter in the alphabet was selected to participate. Additionally, each site enrolled 50 community-based children who were not orphaned or abandoned at baseline as a comparison group.

### Data collection protocol

As previously published [22,26], the following describes the procedures of data collection relevant to this analysis. Children and each of their self-identified primary caregivers were contacted and interviewed twice per year for up to 3 years. Baseline and annual follow up surveys collected data on numerous characteristics including the child’s exposure to traumatic events, symptoms of emotional and behavioral difficulties, cognitive development, and educational attainment. Additionally, caregivers reported on household socioeconomic characteristics. Ethical approval was obtained from the Duke University Institutional Review Board (IRB) and from local and national IRB’s in each participating country.

The primary measures utilized in this study include child self-reports of emotional difficulties, tests assessing cognitive abilities, and child and caregiver reports of exposure to potentially traumatic events, all reported at baseline, and at 12-month, 24-month and 36-month follow ups. The measures used for trauma, emotional difficulties and cognitive development were previously validated for use across cultures (see below) and field-tested using pilot interviews.

### Study measures

#### Measuring cognitive development: kaufman assessment battery for children and the california verbal learning test

The KABC-II is an individually administered test of intelligence and achievement that was developed with the intention of “building in sensitivity to preschoolers, minorities and exceptional populations” [28]. Three nonverbal subtests from the Second Edition of the Kaufman Assessment Battery for Children (KABC-II), Hand Movements, Triangles and Pattern Reasoning, were assessed at annual child interviews in the POFO study. The KABC was chosen because it is one of the most frequently used tests of learning ability internationally. The three non-verbal subtests were chosen to be used across the five countries as they are less dependent on language differences.

The California Verbal Learning Test (CVLT-C) is a test of verbal memory, used by POFO researchers as an indicator of memory, attention and motivation [15]. POFO interviewers modified the memory list, referred to here as the Market List (ML). Locally relevant lists were developed at each site that contained 15 items a child might see at their respective markets. The test required children to encode and store information in order to repeat back what was read to them. The Market List was chosen based on observed variability of children’s engagement with the tests during pilot work in East Africa, suggesting that a tool that reflects motivation and attention would be an important addition to the learning tasks on the KABC-II [15].

Previous analyses by POFO researchers validated these tests as measures of learning and performance for children living in LMIC [15]. The findings of this previous analysis provided support that across the five countries, the subtests functioned as one would expect measures of learning to function, that is, raw scores increased with chronological age. These tests were also strongly associated with years in school for age. Hence, the KABC II scores used here can be seen as an effective tool for measuring learning, which also reflects experience in the learning environment [15].

This analysis used the highest of the three KABC test standard scores (called *topscore*) for each child at each round as the primary outcome measure for cognitive development. This measure represents the best the child was able to do across the three subtests when tested by the interviewer. Standard scores of the KABC II range between 0 and 19, and each subtest has a mean of 10 and a standard deviation of 3 [28]. In this analysis, scores were scaled to US age standards to enable comparison across children and these five settings. The average number of items recalled in the first three repetitions on the Market List was used as an ancillary measure of learning, attention, and motivation.

#### Measuring psychosocial well-being and emotional difficulties: the strengths and difficulties questionnaire

The Strengths and Difficulties Questionnaire (SDQ) is a behavioral screening tool, designed for children ages 4-16, that measures psychosocial well-being across five dimensions: (1) emotional symptoms, (2) conduct problems, (3) hyperactivity/inattention, (4) peer relationship problems, and (5) prosocial behavior. Each subscale has 5 items, scored on a 3-point Likert scale (0-2). The four difficulties subscales add up to a Total Difficulties Score, while the fifth subscale provides assessment of prosocial behavior. POFO researchers chose the Strengths and Difficulties Questionnaire “for its brevity, its psychometric properties, and its frequent use in other international studies” [22]. The questionnaire can be completed in two versions, either by parents, teachers or caregiver report, or, for children ages 11 and older, by self-report [29]. With scoring from 0-2 on each individual item, the Total Difficulties scale ranges from 0 - 40. This analysis used the Total Difficulties score as a continuous variable, rather than using a clinical cutoff, which is not available across these sites. The validity of the self-reported Total Difficulties scale has been assessed and confirmed in multiple contexts (Cronbach’s alpha ranging from 0.73-0.89), indicating that the scale itself is internally valid [1]. In the POFO sample, Cronbach’s alpha was 0.73. These analyses used the Total Difficulties Score self-reported by the child as the primary measure of emotional difficulties. Limiting the SDQ self report to ages 11 and older is in line with the recommendation of the SDQ^b^.

#### Measuring adverse childhood experiences: the life events checklist

This analysis used the Life Events Checklist, first created by the National Center for Posttraumatic Stress Disorder (PTSD) to aid in the diagnosis of post traumatic symptoms [30]. This checklist, which inquires about exposure to potentially traumatic events such as natural disasters, witnessing someone being hurt or killed, experiencing physical or sexual abuse, or being forced to leave home, is one of the most commonly used research instruments to evaluate exposure to trauma across countries and cultures [31]. Caregivers and children were independently asked at each interview whether the child had ever witnessed or experienced each of 21 types of events. A child was counted as having experienced an event if either the caregiver or the child reported it. As described previously, four categories of events were excluded from this analysis [22].^c^ A cumulative traumatic exposure variable was generated for this analysis, which sums the total count of up to 17 different traumatic event categories reported through any given round.

#### Additional covariates: household wealth, caregiver illiteracy, and relationship to the child

An asset checklist and other elements from the Demographic and Health Surveys (DHS)^d^ of each site were used to derive a wealth index score for each participating household [32-36]. Wealth index scores are continuous, standardized for comparability with wealth index scores in each country’s DHS, and indicate greater affluence as the score increases. Caregiver illiteracy was assessed based on a literacy test administered at the time of each survey. Caregivers unable to read four short sentences in the local language were classified as illiterate. The child’s relationship to the caregiver (parent versus non-parent) and orphan status (single or double orphan v. abandoned) were included in the analysis.

#### Analyses

A linear regression model was estimated to describe the relationship between emotional difficulties (SDQ Total Difficulties score) and various explanatory variables, including orphan status, exposure to potentially traumatic events, and household wealth. Additional linear regression models with the KABC *topscore* and Market lists as dependent variables were used to estimate the association between emotional difficulties and cognitive development. These models controlled for age, gender, orphan status, wealth and caregiver illiteracy, and the number of prior administrations of the KABC test to account for child learning over time. Models analyzed up to four time points for each child cross-sectionally; each model specification was run separately by site and jointly for all sites.

Child-level fixed effects models were estimated to describe the relationship between the SDQ Total Difficulties score and cognitive outcomes while controlling for time invariant characteristics of children that may affect outcomes. Models were run jointly across all sites and controlled for age as the only other observed time varying characteristics expected to be associated with the child’s cognitive development during the study period. Additional models, run for sensitivity analysis, evaluated whether the association between emotional difficulties and cognitive outcomes differed by caregiver type (parent versus non-parent), OAC status, or study site. Effect estimates for subgroups were calculated as linear combinations of SDQ main effects and interactions with the respective indicator variables for each subgroup. Two additional fixed effects model analyzed the association of cognitive outcomes with the four SDQ subscales (which comprise the SDQ Total Difficulties score) and with caregiver reports of the SDQ.

All models were estimated with robust standard errors to account for error correlations within sites and between multiple observations from each child. Child-level fixed effects models accounted for clustering at the level of the child. Weights were constructed to account for differences in the number of children and their age and gender distributions across study sites and were used in all models.

#### Attrition

To evaluate the extent to which attrition may have biased our estimates, bivariable logistic regression models of baseline characteristics analyzed whether children who left the study differed significantly from those who stayed.

## Results

### Descriptive statistics

Table 1 shows descriptive statistics at baseline, including proportion and frequency of OAC status, frequency of caregiver type (parent versus non-parent) and the mean and standard deviation of performance on the KABC II, child self-reported emotional difficulties, mean count of exposures to types of potentially traumatic events, and other characteristics.

**Table 1:** Descriptive statistics at baseline

	**Cambodia**	**Ethiopia**	**Hyderabad**	**Kenya**	**Nagaland**	**Tanzania**	**ALL SITES**
Topscore at baseline							
Mean (SD)	7.4 (2.57)	7.6 (2.57)	6.96 (1.86)	7.43 (2.68)	6.71 (2.15)	6.61 (2.27)	7.12 (2.40)
N	300	300	300	300	279	302	1781
Market list at baseline							
Mean (SD)	7.68 (2.59)	7.71 (1.97)	6.47 (1.78)	7.46 (2.17)	6.5 (2.40)	7.48 (2.06)	7.23 (2.23)
N	297	300	299	297	266	301	1760
Total difficulties self-reported at baseline							
Mean (SD)	14.22 (5.49)	10.0 (4.49)	13.74 (4.89)	8.41 (4.15)	8.25 (3.37)	5.27 (3.74)	10.17 (5.68)
N	122	34	50	69	68	85	428
Total difficulties caregiver reported at baseline							
Mean (SD)	13.26 (4.89)	12.81 (6.05)	13.04 (5.30)	9.63 (4.11)	8.77 (3.89)	6.90 (4.80)	10.71 (5.47)
N	292	295	253	300	259	300	1699
Exposure to potentially traumatic events at baseline							
Mean (SD)	2.93 (1.68)	2.64 (1.68)	2.87 (1.95)	0.87 (1.32)	0.81 (0.94)	2.54 (1.08)	2.12 (1.74)
N	300	297	252	299	263	300	1711
Wealth index mean (SD)	0.98 (0.50)	0.97 (0.56)	1.01 (0.36)	1.00 (0.69)	0.99 (0.55)	1.00 (0.62)	0.99 (0.56)
N	258	265	299	281	267	286	1656
Frequency of OAC status at baseline N (%)							
Single orphan	178 (59.33)	161 (53.67)	151 (50.33)	207 (69)	205 (73.48)	178 (58.94)	1080 (60.64)
Double orphan	60 (20)	60 (20)	22 (7.33)	31 (10.33)	17 (6.09)	67 (22.19)	257 (14.43)
Abandoned	13 (4.33)	33 (11)	78 (26)	15 (5)	7 (2.51)	6 (1.99)	152 (8.53)
Non orphans	49 (16.33)	46 (15.33)	49 (16.33)	47 (15.67)	50 (17.92)	51 (16.89)	292 (16.4)
Total N	300	300	300	300	279	302	1781
Caregiver type N (%)							
Parent	135 (45)	117 (39)	96 (32)	110 (36.67)	90 (32.26)	131 (43.38)	679 (38.12)
Non-parent	165 (55)	183 (61)	204 (68)	190 (63.33)	189 (67.74)	171 (56.62)	1102 (61.88)
Total N	300	300	300	300	279	302	1781
Illiterate caregiver N (%)	95 (46.57)	100 (43.48)	173 (64.07)	174 (63.5)	116 (44.27)	52 (22.71)	710 (48.33)
N	204	230	270	274	262	229	1469
Age Mean (SD)	9.41 (1.97)	9.05 (1.76)	9.13 (1.52)	9.25 (1.84)	9.07 (1.86)	9.43 (1.90)	9.22 (1.82)
N	300	300	300	300	279	302	1781
Gender N (%)							
Male	149 (49.67)	160 (53.33)	149 (49.67)	155 (51.57)	160 (57.35)	157 (51.99)	930 (52.22)
Female	151 (50.33)	140 (46.67)	151 (50.33)	145 (48.33)	119 (42.65)	145 (48.01)	851 (47.78)
Total N	300	300	300	300	279	302	1781

Single orphans constitute the largest group within the sample (60.6%). While the average *topscore* at baseline is similar across sites (ranging from 6.6 to 7.6), there is more variation by site on the average level of self-reported emotional difficulties (range 5.3 to 14.2), with Cambodia and Hyderabad reporting the highest average scores. The average self-reported Total Difficulties score was 10.2 for the entire sample at baseline. On average, children had experienced 1.7 types of potentially traumatic events, in addition to their orphaning or abandonment. Nagaland and Kenya had the lowest average levels of reported exposure to potentially traumatic events at baseline.

### Predicting emotional difficulties

Table 2 describes the relationship between the self-reported Total Difficulties score among children 11 and older and a host of explanatory variables, including age, gender, orphan status, wealth, and exposure to potentially traumatic events.^e^

**Table 2:** OLS estimate of total difficulties score predicted by orphan status

**VARIABLES**	**Cambodia total difficulties**	**Ethiopia total difficulties**	**Hyderabad total difficulties**	**Kenya total difficulties**	**Nagaland total difficulties**	**Tanzania total difficulties**	**ALL SITES**§ **total difficulties**
Exposure to potentially traumatic events	0.25	0.12	0.17	0.33**	-0.27	0.60**	0.36**
	(0.14)	(0.12)	(0.1)	(0.07)	(0.15)	(0.21)	(0.06)
Wealth index	-0.63	-0.47	-0.53	-0.14	-0.69**	-0.44	-0.49**
	(0.47)	(0.34)	(0.36)	(0.22)	(0.26)	(0.46)	(0.09)
Single orphan	1.2	1.40**	0.56	0.12	-1.09*	4.52**	0.87
	(0.72)	(0.53)	(0.37)	(0.39)	(0.47)	(0.55)	(0.8)
Double orphan	-0.01	0.72	0.98	-0.99	-1.75*	4.88**	0.68
Abandoned	-0.18	0.65	0.19	0.14	-1.53*	3.18	0.35
	(1.15)	(0.75)	(0.39)	(0.86)	(0.66)	(2.22)	(0.60)
Non-parent caregiver	-0.09	0.72	0.25	0.36	0.24	-1.26	0.04
	(0.55)	(0.50)	(0.32)	(0.40)	(0.34)	(0.67)	(0.20)
Age in months	-0.04**	-0.03**	-0.04**	-0.02*	-0.01	0	-0.03*
	(0.01)	(0.01)	(0.01)	(0.01)	(0.01)	(0.02)	(0.01)
Female	-1.16*	-0.02	-1.01**	-0.03	-1.09**	-0.08	-0.61*
	(0.48)	(0.36)	(0.27)	(0.30)	(0.27)	(0.49)	(0.23)
Constant	19.11**	12.61**	16.78**	10.64**	11.81**	0.69	15.34**
	(2.04)	(1.84)	(2.10)	(1.34)	(1.21)	(2.32)	(2.10)
Observations	438	410	656	573	550	584	3,211
R-squared	0.11	0.18	0.18	0.16	0.09	0.16	0.28

Robust standard errors in parentheses **p<0.01, *p<0.05.

§Model also included site level and round level fixed effects (not shown).

Several factors were associated with children’s emotional difficulties, including child age and gender, exposure to trauma, and household wealth; however, associations varied across sites. In Cambodia, Ethiopia, Hyderabad and Kenya, Total Difficulties were lower for older children. Female gender was associated with lower levels of difficulties in Cambodia, Hyderabad, and Nagaland; wealth was negatively associated with higher levels of emotional difficulties in Nagaland. In two of the six sites, Kenya and Tanzania, exposure to potentially traumatic events was significantly and positively associated with higher levels of emotional difficulties.

There were mixed results for orphan status. Being a single orphan was associated with higher emotional difficulties relative to non-orphans in 2 sites, and being a double orphan was associated with higher emotional difficulties in one site. However orphaned and abandoned children in Nagaland reported lower rates of emotional difficulties relative to comparison children. While the wealth index, exposure to potentially traumatic events, gender and age were statistically significant when looking jointly across “All Sites”, no orphan status held significant associations with emotional difficulties in this specification.

### Predicting cognitive development

Tables 3 and 4 show ordinary least squares (OLS) and fixed effects regressions of emotional difficulties predicting cognitive development. Since exposure to potentially traumatic events was highly correlated with emotional difficulties (rho = 0.33; also see Table 2), exposure to trauma was not included in these models.

**Table 3:** OLS estimate of topscore predicted by total difficulties

**VARIABLES**	**Cambodia**	**Ethiopia**	**Hyderabad**	**Kenya**	**Nagaland**	**Tanzania**	**ALL SITES**§
	**Topscore**	**Topscore**	**Topscore**	**Topscore**	**Topscore**	**Topscore**	**Topscore**
Total difficulties	-0.09**	-0.09*	-0.01	-0.07**	-0.11**	-0.08**	-0.09**
	(0.03)	(0.04)	(0.02)	(0.03)	(0.02)	(0.01)	(0.01)
Age in months	-0.01	0.01	-0.01*	0	-0.03**	0	-0.01
	(0.01)	(0.01)	(0.01)	(0.01)	0.00	0.00	(0.01)
Female	-1.20**	-0.24	0.07	-0.68**	-0.14	0	-0.36
	(0.25)	(0.22)	(0.17)	(0.22)	(0.15)	(0.14)	(0.17)
Single orphan	-0.09	-0.48	-0.26	0	0.09	-0.24	-0.1
	(0.34)	(0.42)	(0.23)	(0.27)	(0.19)	(0.20)	(0.09)
Double orphan	-0.56	-0.41	-0.51	-0.45	0.43	0.08	-0.08
	(0.44)	(0.46)	(0.34)	(0.41)	(0.41)	(0.23)	(0.14)
Abandoned	0.36	-0.39	-0.17	0.28	0.18	0.85	-0.03
	(0.83)	(0.55)	(0.25)	(0.57)	(0.40)	(0.66)	(0.11)
Illiterate caregiver	-0.01	-0.13	0	-0.56*	0.02	-0.36*	-0.16
	(0.28)	(0.22)	(0.18)	(0.23)	(0.15)	(0.18)	(0.09)
Wealth index	0.80**	-0.03	0.22	0.46*	0.1	0.13	0.31*
	(0.26)	(0.21)	(0.24)	(0.18)	(0.14)	(0.11)	(0.11)
Constant	10.00**	7.12**	8.69**	7.96**	10.05**	7.35**	9.07**
	(1.26)	(1.31)	(1.08)	(1.19)	(0.69)	(0.75)	(0.52)
Observations	371	390	675	569	543	553	3,101
R-squared	0.14	0.09	0.4	0.07	0.22	0.22	0.34

Robust standard errors in parentheses **p<0.01, *p<0.05.

§Model also included site level and round level fixed effects (not shown) Table 3. OLS Estimate of Topscore Predicted By Total Difficulties.

**Table 4:** Fixed effects estimate of topscore predicted by total difficulties

			**Sensitivity analysis**				
		**Main model**	**Model 2**	**Model 3**	**Model 4**	**Model 5**	**Model 6**
		**SDQ**	**SDQ**	**SDQ**	**Self report**	**Self report**	**Self report**
		**Self report**	**Subscales**	**CG report**	**by OAC status**	**by CG type**	**by site**
SDQ self report	Total difficulties	-0.09**					
		(0.01)					
	Emotions		-0.08**				
			(0.03)				
	Conduct		-0.04				
			(0.04)				
	Hyperactivity		-0.12**				
			(0.03)				
	Peer relations		-0.13**				
			(0.03)				
SDQ caregiver report total difficulties				-0.07**			
				(0.01)			
Orphan status	Non-orphan				-0.04		
					(0.04)		
	Single orphan				-0.09**		
					(0.01)		
	Double orphan				-0.11**		
					(0.02)		
	Abandoned				-0.13**		
					(0.04)		
Caregiver	Parent					-0.10**	
						(0.01)	
	Non-parent					-0.09**	
						(0.01)	
Study site	Cambodia						-0.00
							(0.02)
	Ethiopia						-0.09**
							(0.04)
	Hyderabad						-0.18**
							(0.03)
	Kenya						-0.09**
							(0.03)
	Nagaland						-0.11**
							(0.03)
	Tanzania						-0.09**
							(0.02)
Observations		3,418	3,418	5,882	3,418	3,327	3,418
R-squared		0.14	0.14	0.13	0.14	0.14	0.15
Number of children		1,445	1,445	1,775	1,445	1,442	1,445

Robust standard errors in parenthesis **p<0.01, *p<0.05.

Estimates from fixed effects linear regression models, controlling for child age, SDQ variable(s), and interactions of OAC status, caregiver type, or study site with SDQ Effects for subgroups calculated as linear combinations of SDQ main effects and interaction effect for the respective indicator variable.

There was a significant negative association between emotional difficulties and the child’s *topscore* on the KABC II tests in five of six study sites (Table 3). There is no evidence that orphan status holds a significant relationship to the child’s *topscore*. Wealth is positive and significant in Cambodia and Kenya. Caregiver illiteracy is associated with lower performance on the KABC II tests, with significant associations in Kenya and Tanzania. Female gender was associated with lower KABC II scores in Cambodia and Kenya.

Table 4 shows the results of a fixed effects model of the relationship between emotional difficulties and cognitive development, thus controlling for all time invariant characteristics within one child. Since most covariates in previous models are considered time invariant, age is the only additional covariate included in the model.

The relationship between emotional difficulties and cognitive development remains negative and significant in the fixed effects estimation (Table 4). In sensitivity analysis, the relationship also holds for three out of the four subscales of the self-reported SDQ (Emotions, Hyperactivity and Peer Relations) as well as the caregiver reported SDQ (Model 3). The negative and significant relationship also holds for most subgroups evaluated in the sensitivity analysis. The relationship between emotional difficulties and cognitive development remains negative and significant for each orphan type (with no significant effect for non-orphans), and across caregiver types (parent versus non-parent). The estimated effect was statistically significant in four out of the six study sites. Effects estimates for the association between SDQ and *topscore* did not differ between single and double orphans (p-value 0.711) or children with parent vs. non-parent caregivers (p-value 0.625; not shown).

The same models as in Tables 3 and 4 were estimated for the CVLT-T Market List to test whether associations held across multiple measures of learning. The correlation between the KABC II *topscore* and the CVLT-T Market List is 0.373, indicating that while there is some shared variance, the two indices still likely capture different aspects of the child’s cognitive development. The relationship between emotional difficulties and scores on the Market List test was negative and significant in 3 of 6 sites for the OLS model. The relationship was also negative and significant across “All Sites” in the fixed effects model, with similar results for the sensitivity analysis as were found with the *topscore* measure. Results are presented in Tables 5 and 6.

**Table 5:** OLS estimate of market list predicted by total difficulties

**VARIABLES**	**Cambodia**	**Ethiopia**	**Hyderabad**	**Kenya**	**Nagaland**	**Tanzania**	**Tanzania ALL SITES**§
	**Market list**	**Market list**	**Market list**	**Market list**	**Market list**	**Market list**	**Market list**
Total difficulties	0.01	-0.07**	-0.02	-0.03	0.04*	-0.20**	-0.09
	(-0.02)	(-0.02)	(-0.02)	(-0.02)	(-0.02)	(-0.01)	(-0.04)
Age in months	0.03**	0.03**	0.02**	0.02**	0.01*	0.01**	0.02**
	(-0.01)	(-0.01)	(0.00)	(-0.01)	(0.00)	(0.00)	(0.00)
Female	-0.01	0.17	0.16	0.17	0.15	0.15	0.05
	(0.20)	(0.15)	(0.11)	(0.15)	(0.11)	(0.13)	(0.03)
Single orphan	0.01	-0.23	0.09	-0.32	0.17	0.3	-0.07
	(0.30)	(0.25)	(0.14)	(0.18)	(0.15)	(0.23)	(0.09)
Double orphan	-0.31	-0.33	0.33	-0.78**	0.23	0.64*	-0.06
	(0.35)	(0.28)	(0.21)	(0.29)	(0.30)	(0.25)	(0.22)
Abandoned	0.22	-0.71*	-0.06	-0.78	0.44	0.43	-0.25
	(0.48)	(0.34)	(0.15)	(0.45)	(0.31)	(0.60)	(0.14)
Illiterate caregiver	0.19	-0.21	-0.23	-0.35*	-0.11	-0.39*	-0.19
	(0.21)	(0.15)	(0.12)	(0.16)	(0.12)	(0.18)	(0.09)
Wealth index	0.48*	0.04	0.07	0.1	0.11	0	0.1
	(0.22)	(0.14)	(0.14)	(0.11)	(0.11)	(0.12)	(0.05)
Constant	4.50**	5.95**	5.07**	6.09**	5.64**	6.88**	8.03**
	(0.98)	(0.89)	(0.72)	(0.80)	(0.52)	(0.61)	(0.97)
Observations	371	390	675	568	542	552	3098
R-squared	0.08	0.16	0.31	0.12	0.04	0.48	0.3

Robust standard errors in parentheses **p<0.01, *p<0.05.

§Model also included site level and round level fixed effects (not shown).

**Table 6:** Fixed effects estimate of market list predicted by total difficulties

			**Sensitivity analysis**				
		**Main model**	**Model 2**	**Model 3**	**Model 4**	**Model 5**	**Model 6**
		**SDQ**	**SDQ**	**SDQ**	**Self report**	**Self report**	**Self report**
		**Self report**	**Subscales**	**CG report**	**by OAC status**	**by CG type**	**by Site**
SDQ self report	Total difficulties	-0.09**					
		(0.01)					
	Emotions		-0.08**				
			(0.02)				
	Conduct		-0.01				
			(0.03)				
	Hyperactivity		-0.08**				
			(0.03)				
	Peer relations		-0.17**				
			(0.03)				
SDQ caregiver report total difficulties				-0.06**			
				(0.01)			
Orphan status	Non-orphan				-0.03		
					(0.02)		
	Single orphan				-0.09**		
					(0.01)		
	Double orphan				-0.10**		
					(0.02)		
	Abandoned				-0.08*		
					(0.03)		
Caregiver	Parent					-0.08**	
						(0.01)	
	Non-parent					-0.08**	
						(0.01)	
Study site	Cambodia						-0.01
							(0.02)
	Ethiopia						-0.04
							(0.03)
	Hyderabad						-0.12**
							(0.02)
	Kenya						-0.01
							(0.02)
	Nagaland						0.03
							(0.03)
	Tanzania						-0.18**
							(0.01)
Observations		3,415	3,415	5,856	3,415	3,324	3,415
R-squared		0.11	0.12	0.13	0.12	0.11	0.16
Number of children		1,445	1,445	1,773	1,445	1,442	1,445

Robust standard errors in parentheses **P<0.01, *p<0.05.

Estimates from fixed effects linear regression models, controlling for child age, SDQ variable(s), and interactions of OAC status, caregiver type, or study site with SDQ Effects for subgroups calculated as linear combinations of SDQ main effects and interaction effect for the respective indicator variable.

### Attrition and missing data

Attrition differed significantly across study sites (p-value 0.00) and children living with parents (single or non-orphan) were less likely to drop out (p-value 0.046). Results in Tables 4 and 6 suggest that the observed associations do not differ between children living with parents and non-parents, thus it is unlikely that differential attrition between children residing with biological parents versus other children substantively biased our estimates. Further, the direction of the observed associations was similar in most study sites. There were no differences in rates of attrition across other relevant factors such as exposure to potentially traumatic events, cognitive development, socioeconomic status, gender, or the level of emotional difficulties.

Using the child self-report measure of the Total Difficulties scale resulted in a number of missing data points at baseline, since children did not self-report on the SDQ until they were 11 years old. To check whether missing data of this nature might change results, cross-sectional analysis was restricted to the 36-month follow up (when most children (84.3%) were old enough to self-report) controlling for baseline characteristics, with little change in results. Given that a) the caregivers of nearly half of all participating children were not their biological parents, b) caregivers may significantly under-report the behavioral effects of traumatic events experienced by children [37], and c) caregivers had been taking care of these children for variable amounts of time, the child self-reported difficulties were considered more accurate than caregiver reports.

## Discussion

This is the first cross-country study to examine the relationship between orphans’ emotional difficulties and their learning. Moreover, conducting such an analysis on a unique sample of vulnerable children who are susceptible to multiple instances of adversity, including trauma, provides evidence of the nature of this relationship within a context of heightened adversity and potentially higher risk of mental health difficulties. This analysis showed that OAC exposure to potentially traumatic events is an important predictor of emotional difficulties, thus supporting previous literature that the number of adverse events matters for risk of mental health difficulties.

The most salient finding was a negative and significant relationship between a child’s emotional difficulties and his/her cognitive development, within and across five of six study sites. Rates of exposure to adverse childhood events among the orphaned and abandoned children in this study were high, and exposure to adverse events, in addition to male gender, and lower household wealth, was associated with significantly higher rates of emotional difficulties. Interestingly, orphan status was only significantly associated with emotional difficulties in some sites and was not predictive of cognitive development, although the negative and significant relationship between emotional difficulties and cognitive development held within each orphan subgroup. These findings may suggest that when a child is more vulnerable to a number of adverse events, it is the context in which a child loses a parent, rather than the loss of a parent alone, that better explains mental health outcomes. Children who are vulnerable from orphaning and have experienced other adversity are at heightened risk of increased emotional difficulties, which is associated with lags in cognitive development. These findings offer insights into the relationship between vulnerable children’s mental health and their ability to participate in and benefit from education.

In the context of multiple adversity, the loss of a parent, or orphan status, does not show statistical significance in the hypothesized relationship between mental health and learning. Nevertheless, the overall findings still underscore the importance of mental health intervention for children who are especially vulnerable. It is likely that the effect of parental loss is further affected by the additional adverse events to which the child is even more vulnerable. No trauma or potentially traumatic event happens in isolation.

Although causality cannot be inferred from this analysis, the results offer insight into the relationship between emotional difficulties and educational outcomes. The fixed effects model controls for unobserved time-invariant differences between children that may be correlated with emotional difficulties and may influence cognitive development. Consequently, the results in Table 4 give us an unbiased estimate of the relationship between a child’s emotional difficulties and his/her cognitive development, controlling for these differences. The consistency of the association between these two factors across multiple contexts and measures and within different subgroups is striking. This underscores the importance of providing trauma support and focusing on the psychosocial development of vulnerable children as a means to alleviate strains inhibiting a child’s learning.

Additionally, the associations found between wealth and emotional difficulties may indicate that interventions aimed at easing households’ resource constraints could help alleviate emotional difficulties in some contexts. One may hypothesize that in less stable, resource poor settings, children who were exposed to trauma may be less likely to recover from emotional difficulties. Interventions on both the child and family levels may work in tandem to improve education outcomes for orphans. If policies and programs can improve economic and emotional stability for both children and families, orphans may have a greater chance of pushing past the challenges of losing a parent and the additional traumas to which orphanhood exposes them. While economic support does not treat mental health difficulties, it may provide families with the resources needed to keep children in school, which may aide their psychosocial development. Such support may improve a child’s chances of overcoming the emotional challenges that are associated with lags in cognitive development. We acknowledge several limitations.

Children’s cognitive development is described by only two measures, an aggregated score summarizing children’s performance on three nonverbal KABC II subtests plus a measure verbal memory and attention based on the CVLT. While ability is likely not to change over time, the child’s performance on the KABC II tests is not only an indication of the child’s ability, but also a variety of other factors associated with motivation, self-confidence, response to authority and the child’s developed non-verbal skillset [1]. We may interpret the association of test scores with the child’s emotional difficulties to be related to changes in these additional factors. While this measure may not singularly isolate the child’s cognitive development, knowledge of the critical role of emotional difficulties in the child’s learning and subsequent academic attainment is an important insight for policies on global support for vulnerable children, including but not limited to those vulnerable due to parental death.

Further, the available data do not offer adequate statistical power to describe the temporal association between the variables of interest, and therefore cannot rule out possibilities of reverse causality. It is possible that the observed relationships are circular and that, even if higher emotional difficulties impede cognitive development, lags in cognitive development in turn may heighten emotional difficulties. With adversity, psychosocial manifestations, and cognitive delays spanning much of the life span of the children in this study, the three year follow-up period does not offer sufficient within-child variation to disentangle these effects. Regardless of the direction of causality, if we know that children who are exposed to more trauma also report more emotional difficulties and perform lower on tests of cognitive ability, interventions to provide emotional support for children living in adversity will help at least one or more of these difficulties. Even though we do not know whether emotional difficulties are driving lags in cognition or the other way around, we still need to address the emotional difficulties, specifically targeting potentially traumatic events.

Finally, the study findings may not be generalizable, as there are many other groups of OAC, including street children, institution-based orphans, and those that live in countries with widely different contexts than those in our sample. These associations may differ in those samples. The variability in the observed associations across the six study sites suggests that associations between trauma, emotional difficulties and cognitive development, and appropriate interventions, are likely context-specific. Nevertheless, this analysis offers new insight into the relationship between psychosocial factors, exposure to adverse childhood experiences, resource constraints and the cognitive development of orphans in wide variety of contexts and diverse settings in Southeast Asia and sub-Saharan Africa.

## Conclusions

This study suggests that increased reports of exposure to potentially traumatic events among orphans and abandoned children are associated with higher emotional difficulties, and increases in emotional difficulties are associated with lags in cognitive development. Hence, exposure to trauma and emotional difficulties comprise important barriers to educational attainment for all such vulnerable children, including orphans. Higher socioeconomic status and better educated caregivers may offer buffers to these difficulties, since they are associated with fewer emotional difficulties and higher performance on tests of cognitive development. Interventions targeting both the psychosocial development of the child, vis a vis their exposure to adverse childhood events, and the socio-economic status of the caregiver may work in tandem to improve educational outcomes for vulnerable children in a more holistic sense. Further, family based interventions to stabilize socioeconomic conditions or increase caregiver education may help overcome psychosocial challenges that otherwise would present as barriers to the child’s educational advancement. Most importantly, psychosocial status may be an important actor in a child’s ability to profit from and stay in school, and one factor that influences this status is a child’s exposure to traumatic and other adverse experiences. These findings may provide a guide to developmental strategies for those working to improve education outcomes for children in less wealthy areas.

### Ethical approval

Ethical approval was provided by the Duke Medicine Institutional Review Board (IRB); the local review boards of SaveLives Ethiopia and Stand for Vulnerable Organization (Addis Ababa, Ethiopia), Sharan (Delhi, India), and Kilimanjaro Christian Medical Centre (Moshi, Tanzania); and national regulatory agencies in all participating countries including the National Ethics Committee for Health Research (Cambodia), the Ministry of Science and Technology (Ethiopia), the Indian Council of Medical Research (India), the Kenya Medical Research Institute (KEMRI), and the National Institute for Medical Research (Tanzania).

### Informed consent

Children under the age of 18 were asked for assent to participate in the study only after the guardian had first given permission for the child to participate, and for the interviewer to speak with the child. The project was explained to the child in an age-appropriate manner, and the child was given the opportunity to ask questions about the project before assent was requested. Children who were not competent (who had moderate to severe learning or mental health disorders) to give assent were not included in the study. Children who previously gave assent as minors were re-consented as adults when they reached age 18.

## Endnotes

^a^While the definition of “orphan” varies across cultures and settings, UNICEF provides a commonly accepted definition for orphan as “a child who has lost one or both parents” (UNICEF 2004).

^b^For more information, see sdqinfo.org.

^c^“Hearing about a family member who has died”, “had a brother or sister die”, “seeing a dead body in town”, and “having a painful or scary medical treatment,” were excluded from the trauma count variable. Almost all children had lost a family member, hence the first event did not add to the variation in the count. For the second event, it was not clear whether the child witnessed the event themselves. For the third event, in some sites, burials are open-casket so all kids had seen these events. For the latter event, event description was not specific enough. It seemed that for some children, this included blood draws or like events that were scary to the child but not comparable to the other categories.

^d^Data was compiled from a variety of country specific DHS data sets. See references for further information on data used. 

^e^Constants in linear regression models represent the estimated mean value for the (hypothetical) reference group for whom the values of all variables in the model are zero.

## Competing interests

The authors declare that they have no competing interests.

## Authors’ contributions

ME conceived of the analysis, analyzed the dataset, conducted the literature review and wrote the manuscript. KW assisted with conceptualization of the analysis models and interpretation of the results, and provided manuscript feedback. JO assisted with conceptualization and implementation of the analysis, data analysis, interpretation of the results, and provided manuscript feedback. KOD selected the measures of emotional difficulties and learning and assisted with study implementation and interpretation of the results. All authors read and approved the final manuscript.

## Pre-publication history

The pre-publication history for this paper can be accessed here:

http://www.biomedcentral.com/1472-698X/14/6/prepub
